# Formation of Catalytically Active Nanoparticles under Thermolysis of Silver Chloroplatinate(II) and Chloroplatinate(IV)

**DOI:** 10.3390/molecules27041173

**Published:** 2022-02-09

**Authors:** Evgeny Filatov, Pavel Smirnov, Dmitry Potemkin, Denis Pishchur, Natalya Kryuchkova, Pavel Plyusnin, Sergey Korenev

**Affiliations:** 1 Nikolaev Institute of Inorganic Chemistry, Siberian Branch, Russian Academy of Sciences, 630090 Novosibirsk, Russia; p.smirnov1@g.nsu.ru (P.S.); denispischur@ngs.ru (D.P.); knatali@ngs.ru (N.K.); plus@niic.nsc.ru (P.P.); korenev@niic.nsc.ru (S.K.); 2 Department of Natural Sciences, Novosibirsk State University, 630090 Novosibirsk, Russia; potema@catalysis.ru; 3 Boreskov Institute of Catalysis, Siberian Branch, Russian Academy of Sciences, 630090 Novosibirsk, Russia

**Keywords:** thermolysis, silver, platinum, solid solution of metals, CO PROX

## Abstract

The thermal behaviour of Ag_2_[PtCl_4_] and Ag_2_[PtCl_6_] complex salts in inert and reducing atmospheres has been studied. The thermolysis of compounds in a helium atmosphere is shown to occur in two stages. At the first stage, the complexes decompose in the temperature range of 350–500 °C with the formation of platinum and silver chloride and the release of chlorine gas. At the second stage, silver chloride is sublimated in the temperature range of 700–900 °C, while metallic platinum remains in the solid phase. In contrast to the thermolysis of Ag_2_[PtCl_6_], the thermal decomposition of Ag_2_[PtCl_4_] at 350 °C is accompanied by significant heat release, which is associated with disproportionation of the initial salt to Ag_2_[PtCl_6_], silver chloride, and platinum metal. It is confirmed by DSC measurements, DFT calculations of a suggested reaction, and XRD. The thermolysis of Ag_2_[PtCl_4_] and Ag_2_[PtCl_6_] compounds is shown to occur in a hydrogen atmosphere in two poorly separable steps. The compounds are decomposed within 170–350 °C, and silver and platinum are reduced to a metallic state, while a metastable single-phase solid solution of Ag_0.67_Pt_0.33_ is formed. The catalytic activity of the resulting nanoalloy Ag_0.67_Pt_0.33_ is studied in the reaction of CO total (TOX) and preferential (PROX) oxidation. Ag_0.67_Pt_0.33_ enhanced Pt nano-powder activity in CO TOX, but was not selective in CO PROX.

## 1. Introduction

The search for new efficient energy sources is an important problem nowadays. One option to solve this problem is to use fuel cells, i.e., devices that convert chemical energy into electrical energy, bypassing the inefficient stage of fuel combustion, which occurs with great losses. Since there is no release of harmful substances into the atmosphere, such elements are eco-friendly, which is very important today due to pressing environment-related issues. Therefore, fuel cells are promising and have attracted the attention of many researchers [1,2].

Catalysts are important components of fuel cells; they increase the efficiency of the latter. Various catalysts can be used for low-temperature fuel cells, but highly dispersed platinum catalysts on carbon are currently amongst the most popular ones.

Platinum is widely used in catalytic processes. It was shown in [3,4] that the activity of platinum in the methanol oxidation reaction depends on the particle size and these changes are not monotonic. The substrate and additives also make a significant contribution to the oxidation of methanol. When used as a substrate of transition metal oxides, the rate of methanol oxidation depends on strong interactions between nanoparticles, platinum, and transition metal oxides [4].

However, the use of pure platinum has its drawbacks, such as a high cost of the metal due to limited reserves and degradation during long-term operation as a part of fuel cells, and poisoning by CO. These problems can be solved by including a cheaper metal, e.g., silver, which helps to achieve the desired effect [5].

Silver itself is capable of catalyzing the oxidation reaction of methanol. This is due to the fact that oxygen binds to silver, thus forming a methoxy group [6]. At the same time, depending on the O_2_/CH_3_OH ratio and temperature, different products can be obtained. For example, when the ratio O_2_/CH_3_OH = 0.39 and temperature 640 °C, formaldehyde is formed with a yield of 91% [7], and at a low ratio and a temperature range of 500–650 °C, methyl formate is mainly formed [8].

In the case of Ag/Pt, such samples have a high thermodynamical stability according to the calculations of Ramirez-Caballero by using the density functional theory [9]. This is a unique property of Ag/Pt bimetallic catalysts which makes them promising for catalytic applications [10,11].

The Ag/Pt catalysts are more active in the selective or complete oxidation of methanol or propylene, in contrast to pure platinum and silver [12,13]. The highest activity is achieved with the composition Ag_0.38_Pt_0.62_ [11]. The use of AgPt as a catalyst to synthesise H_2_O_2_ improves the selectivity up to 50%, unlike pure platinum, which yields only 6% [14]. The Ag_2_Pt/Al_2_O_3_ catalyst also shows a high activity when compared to monometallic analogues during CO oxidation under ambient temperature [15]. This is due to the fact that H_2_O and OH bind principally to Ag atoms in the presence of Pt, while the adsorbed OH on Ag atoms oxidizes the adsorbed CO on the Pt surface [16].

Due to the similarity of the lattice parameters of Ag and Pt, they are capable of forming AgPt alloys [17,18]. Moreover, Ag promotes the activation of surface-active centres due to the fact that it can modify the electronic structure [19,20]. Ag promotes an increase in photochemical activity due to the large specific surface area of silver and its unusual electronic properties [21]. It was shown in [22] that AgPt deposited on N-doped graphene quantum dots (N-GQD/AgPt HND) showed a 21-fold improvement in catalytic properties compared to commercial Pt/C. It was also shown that under illumination with visible light, the electrocatalytic activity of N-GQD/AgPt in relation to the oxidation of methanol showed 1.7 times more than without treatment with visible light.

Depending on the morphology and composition, AgPt solid solution shows different chemical and physical properties. The use of thin AgPt films with a nano-fern structure as a substrate increases the sensitivity of creatine molecules in the surface-enhanced Raman scattering (SERS). In the future, AgPt thin films can be potentially implemented in the monitoring of environmental pollution, food quality, safety, and medical applications [23,24]. Furthermore, AgPt nanoparticles with a nano-fern structure show high catalytic and selective activity in the acetone hydrogenation reaction to produce isopropyl alcohol, unlike pure platinum and silver [25].

There is work underway to study the biological effect of AgPt nanoparticles. Thus, bimetallic nanoalloys (AgPt NP) with different metal composition from Ag_10_Pt_90_ to Ag_90_Pt_10_ are considered in [26] with an increment of 20 mol.%. AgPt was found to demonstrate a combination of properties of the simple metals: it shows antimicrobial activity associated with Ag and osteo-promotive effect associated with Pt, as well as inhibiting osteoclastogenesis. This combined effect of AgPt NP is valuable in the development of new antimicrobial and osteo-promotive supplements for biomaterials that can support bone regeneration. The stability of the synthesised nanoalloys is also a significant aspect. As shown in [27], the stability of Ag/Pt clusters decreases with increasing Pt content depending on the cluster composition. The nanoalloy stability increases in the following sequence: 5.1 < 3.6 < 2.5 < 1.6 of the Ag/Pt molar ratio. The cluster’s stability was maintained for the Ag/Pt molar ratio of 0.4–5.1. There have been two particularly stable compositions identified with Ag/Pt ratios of 1.6 and 0.4.

Complex compounds of platinum metals are a good precursor for the synthesis of solid solutions of metals. The thermal properties of hexa- and tetra-chloroplatinic salts of ammonium and alkali metals have been studied in detail [28]. Alkali metal tetra-chloroplatinate(II) is characterised by a disproportionation reaction in the temperature range of 200–400 °C, leading to the formation of Pt, hexa-chloroplatinate(IV), and alkali metal chloride [29]:

2 M_2_[PtCl_4_] = Pt + M_2_[PtCl_6_] + 2 MCl.

Therefore, this work is devoted to the synthesis of Ag_2_[PtCl_6_] and Ag_2_[PtCl_4_] compounds—precursors of the bimetallic nanoalloys with precisely assigned ratio of metals—their thermal properties in inert and reducing atmospheres. Ag_0.67_Pt_0.33_ nanoalloy which is final product of the precursor compounds thermolysis has been studied in the reactions of preferential (PROX) and total (TOX) CO oxidation. Its formation was investigated upon heating up to 614 °C by in situ X-ray diffraction.

## 2. Results and Discussion

### 2.1. Photo Stability of Complex Compounds

Silver salts are well known to be unstable and to decompose under the daylight. We conducted an experiment to determine the photo stability of the synthesised salts: some amount samples of Ag_2_[PtCl_4_] and Ag_2_[PtCl_6_] were placed in a desiccator with P_2_O_5_ in a dark place, while others were left in the light. No mass changes were observed for two months, and no formation of new phases. Furthermore, no decrease in the intensity of the reflections of the initial salts was detected on the diffraction pattern as well.

The additional UV–Vis and PL experiments are described in the Supplementary Materials (S2).

Thus, we can talk about the photo stability of the synthesised compounds in relation to light at least of the Ag_2_[PtCl_6_].

### 2.2. Thermal Properties of Ag_2_[PtCl_4_] in a Inert Atmosphere

**Figure 1:**
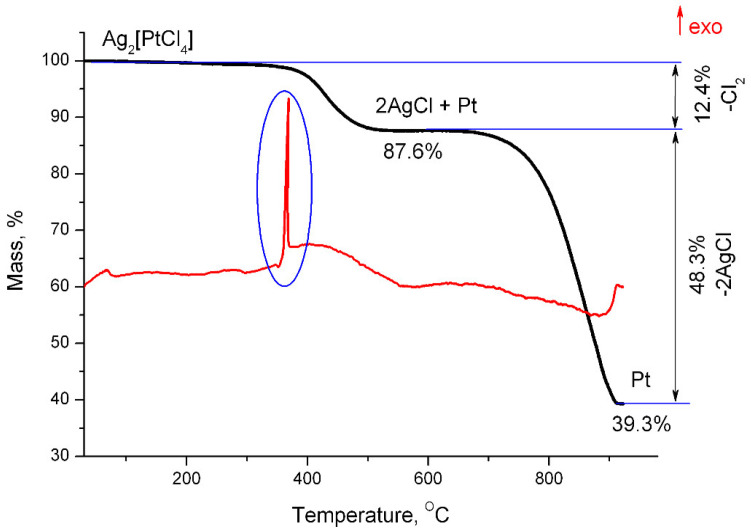
Mass loss curves and differential thermal analysis of Ag_2_[PtCl_4_] in a helium atmosphere.

A total of 12.4% of the mass of the substance is lost during the thermolysis of Ag_2_[PtCl_4_] in an inert atmosphere (helium) in the temperature range of 340–515 °C, which corresponds to the removal of the Cl_2_ molecule, and 87.6% of the initial mass remains in the solid, which corresponds to 2 AgCl and Pt (Figure 1). This assumption is confirmed by X-ray phase analysis.

It is noteworthy that intense heat is released at a temperature of 365 °C (at the very beginning of the decomposition of the initial compound) which could be associated with a phase transition or another process that is not accompanied by a change in mass.

**Figure 2:**
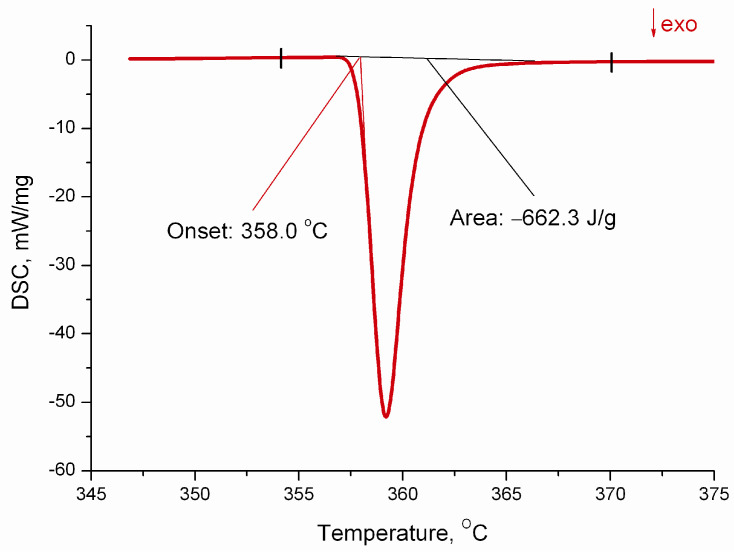
Differential scanning analysis curve for Ag_2_[PtCl_4_].

Samples of the complex Ag_2_[PtCl_4_] weighing 5–14 mg were investigated in the temperature range from 27 to 380 °C. The thermal anomaly with the exo-effect of 662 ± 12 J/g or 366 ± 7 kJ/mol corresponding to the decomposition process at T_onset_ = 358 ± 1 °C was observed on the temperature dependence of the DSC signal (Figure 2). Irreversibility and completion of the decomposition process was checked by reheating of samples after the first measurement.

**Figure 3:**
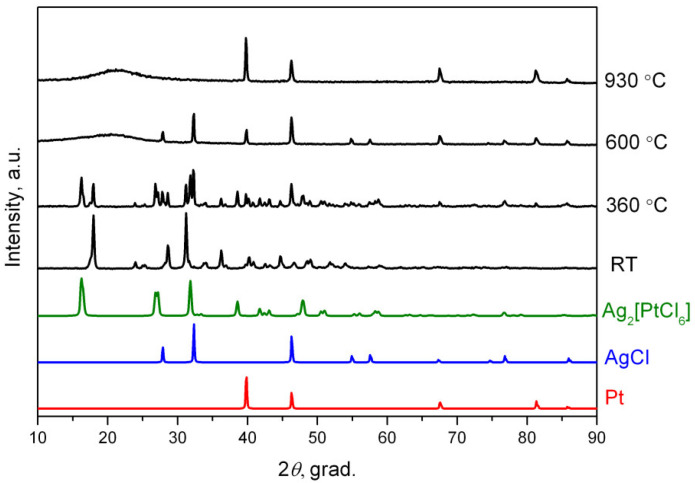
Diffraction patterns of Ag_2_[PtCl_4_] and its thermolysis products in a helium atmosphere at different temperatures. RT-room temperature.

The data of X-ray phase analysis show there is a disproportionation of platinum(II) with the formation of metallic platinum and silver hexachloroplatinate(IV) and AgCl at this stage (Figure 3):

2 Ag_2_[PtCl_4_] = Ag_2_[PtCl_6_] + Pt + 2 AgCl

In order to confirm this assumption, the model complexes were optimised using the Density Functional Theory (DFT). The calculated enthalpy of the proposed reaction gives good agreement with the experiment (3.6 eV or 345 kJ/mol).

In total, 48.3% of the mass is lost in the temperature range of 650–917 °C, which corresponds to AgCl sublimation, and 39.3% of the substance remains, which corresponds to metallic platinum and apparently to the remaining silver chloride (2% of mass). These calculations were also confirmed by X-ray phase analysis (Figure 3).

### 2.3. Thermal Properties of Ag_2_[PtCl_4_] in a Hydrogen Atmosphere

**Figure 4:**
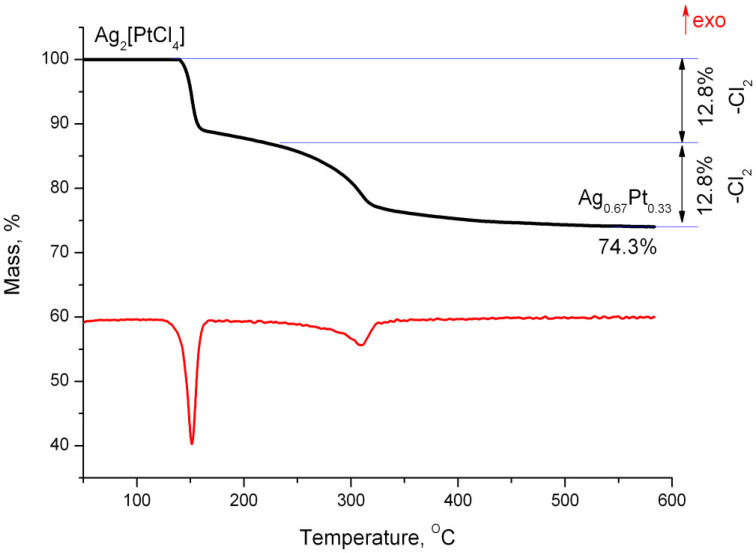
Mass loss curves and differential thermal analysis of Ag_2_[PtCl_4_] in a hydrogen atmosphere.

Two poorly separated stages occur during the thermolysis of Ag_2_[PtCl_4_] in a reducing atmosphere in the temperature range of 100–350 °C. According to the mass loss calculations, these stages involve removal of chlorine molecules (Figure 4). According to X-ray phase analysis, the final product is an FCC phase with the following unit cell parameter: *a* = 4.024(4) Å, *V/Z* = 16.3 Å^3^, crystallite size of 4–6 nm, which corresponds to the composition of Ag_0.67_Pt0._33_ (S3).

### 2.4. Thermal Properties of Ag_2_[PtCl_4_] in a Inert Atmosphere

**Figure 5:**
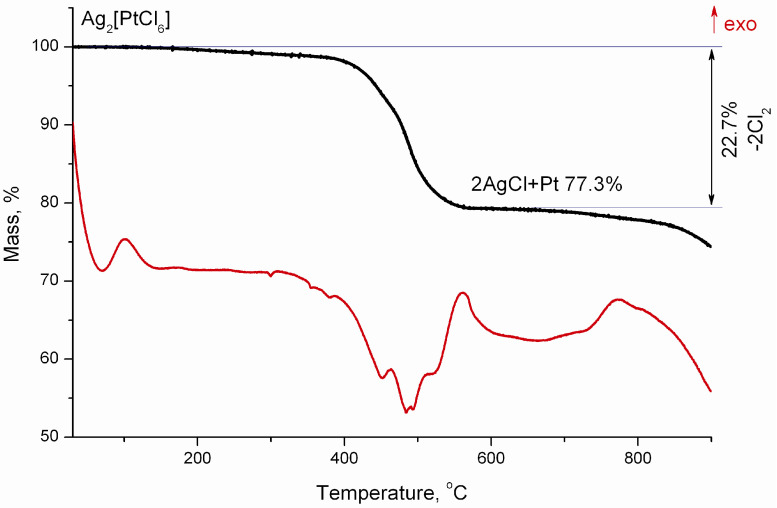
Mass loss curves and differential thermal analysis of Ag_2_[PtCl_6_] in a helium atmosphere.

The behaviour of Ag_2_[PtCl_6_] during thermolysis in an inert atmosphere (helium) is similar to that of Ag_2_[PtCl_4_] with the only difference being a lack of intense heat release at the beginning of decomposition. There is a loss of 22.7% of the mass in the temperature range 370–560 °C, which corresponds to the elimination of two molecules of chlorine, and the remaining mass of 77.3% corresponds to a mixture of platinum and silver chloride in the ratio of 1:2, respectively (Figure 5 and Figure S4).

Above 760 °C, further mass loss occurs which corresponds to the sublimation of silver chloride, while only platinum metal remains in the solid residue.

### 2.5. Thermal Properties of Ag_2_[PtCl_6_] in a Hydrogen Atmosphere

**Figure 6:**
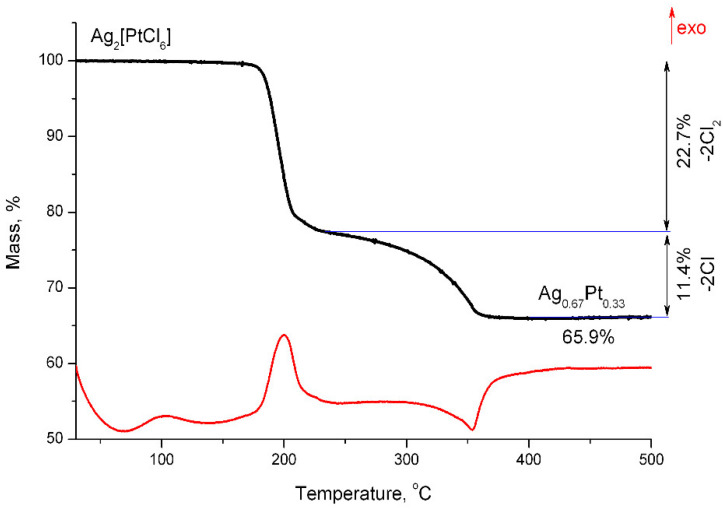
Mass loss curves and differential thermal analysis of Ag_2_[PtCl_6_] in a hydrogen atmosphere.

The first stage of Ag_2_[PtCl_6_] thermolysis in a reducing atmosphere occurs in the temperature range of 170–260 °C, similar to decomposition in a helium atmosphere: 22.7% of the mass is lost, which corresponds to two molecules of chlorine, and the remaining mass of 77.3% corresponds to a mixture of platinum and silver chloride in the ratio 1:2, respectively (Figure 6).

In the temperature range of 260–360 °C, 11.4% of the mass is lost, which corresponds to two Cl atoms; thus, the recovery of silver chloride and the formation of a solid solution of silver and platinum in the ratio of 2:1 occur at this stage.

**Figure 7:**
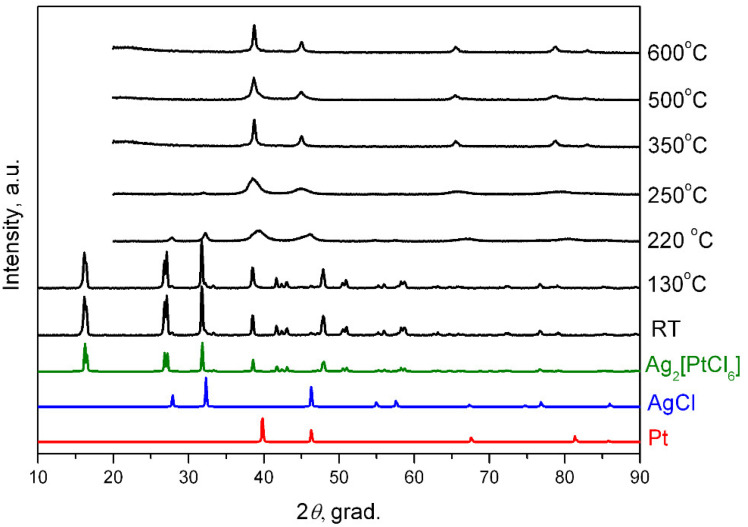
Diffraction patterns of Ag_2_[PtCl_6_] and its thermolysis products in a hydrogen atmosphere at various temperatures. RT- room temperature.

According to X-ray diffraction data (Figure 7), the initial compound remains unchanged when heated to 130 °C, while at 220 °C wide peaks are observed on the diffraction pattern from the platinum phase FCC and AgCl only. An increase in temperature to 250 °C leads to a decrease in the intensity of AgCl reflections, a simultaneous increase in the intensity of the FCC phase, and a shift of the reflections towards smaller angles, which is associated with atoms of silver entering the structure of platinum and forming a solid solution.

**Figure 8:**
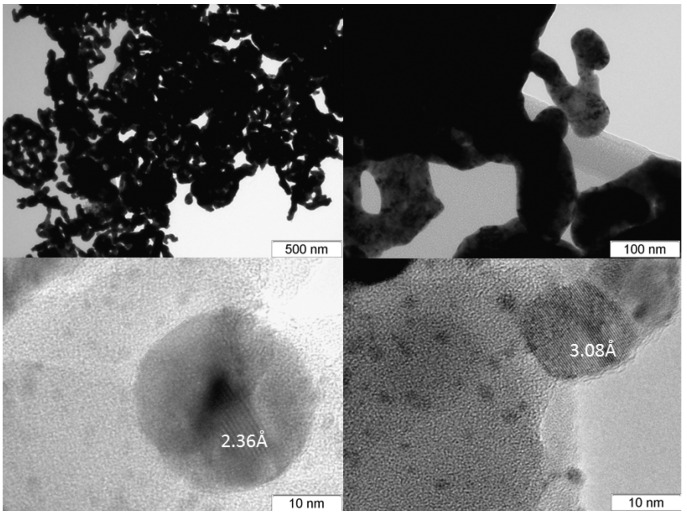
Micrographs of a sample obtained by decomposition of Ag_2_[PtCl_6_] in a hydrogen atmosphere.

At the end point of thermolysis (350 °C), the sample is an FCC phase with the unit cell parameter: *a* = 4.012(4) Å, *V/Z* = 16.2 Å^3^, crystallite size of 3–4 nm as per the XRD data, which corresponds to the composition of Ag_0.67_Pt_0.33_ according to the calibration line. Further heating causes an increase in the crystallite size to 4–6 nm (500 °C). At the same time, a peak asymmetry appears on the diffraction patterns, which is characterised by an increase in the half-width from large angles. The value *V/Z* here increases to 16.4 Å^3^, which may indicate the beginning of solid solution stratification, since the resulting nanoalloy is metastable according to the phase diagram [30]. This assumption is confirmed by EDAX analysis at various points of the sample; the relative content of silver and platinum varies from 48:52 to 96:4 at.%. Thus, a sample obtained in a hydrogen atmosphere at 500 °C includes porous branched agglomerates (Figure 8). Particles with distinguishable observed interplanar distances can be seen in HRTEM images, which correspond at different points to particles of different composition, from pure silver to a solid solution of Ag_0.25_Pt_0.75_.

The sample obtained at 600 °C is an FCC phase with the unit cell parameter: *a* = 4.012(4)Å, *V/Z* = 16.2 Å^3^, and crystallite size of 7–13 nm, which, as for the sample at 350 °C, corresponds to Ag_0.67_Pt_0.33_ along the calibration line. The peak profile is symmetrical, which indicates a lack of the solid solution stratification.

In situ synchrotron studies were conducted to better understand the processes occurring during the thermolysis of Ag_2_[PtCl_6_] in a hydrogen atmosphere.

### 2.6. In Situ X-ray Diffraction Study of the Ag_2_[PtCl_6_] Thermolysis in a Hydrogen Atmosphere

**Figure 9:**
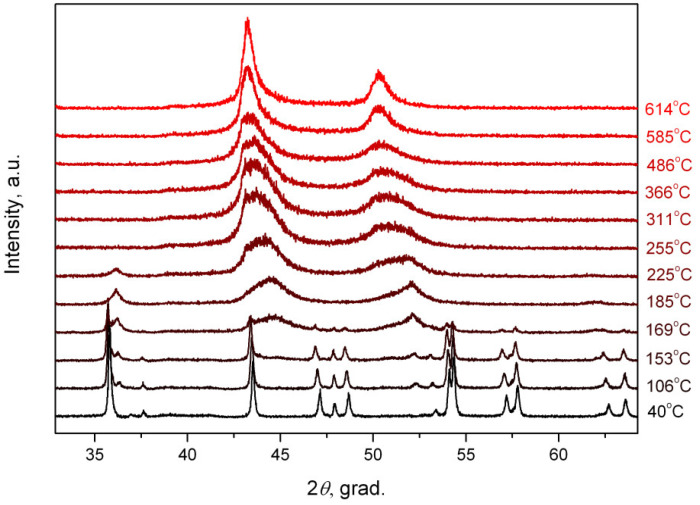
In situ X-ray diffraction of Ag_2_[PtCl_6_] products thermolysis in a hydrogen atmosphere (*λ* = 1.720 Å).

Heating in the in situ mode shows that AgCl peaks (36.3 and 52.3° 2*θ*) already appear in the diffraction pattern (Figure 9) at 106 °C, in addition to the peaks of the initial complex compound. As the temperature increases, the intensity of the AgCl peaks increases and reaches a maximum at 185 °C, while the intensity of the peaks of the initial complex compound Ag_2_[PtCl_6_] decreases. When the temperature reaches 169 °C, the background of the diffraction pattern rises in the area of the angles corresponding to the FCC reflections of the platinum metal phase, while the peaks of the initial compound are almost invisible. At 185 °C, the intensity of the FCC phase peaks increases; an asymmetry of the peaks is manifested at the same time—a shoulder appears from the side of smaller angles, which indicates the formation of metallic silver particles.

With a further temperature increase, AgCl is completely reduced to metallic silver (255 °C) and a set of solid solutions is formed in the Ag–Pt system, as supported by wide peaks with a truncated maximum in the diffraction pattern. Furthermore, the maximum is shifted towards smaller angles up to 614 °C, which is evidence of homogenisation and the formation of a solid solution with a clearly defined ratio of metals.

The sample obtained at 614 °C is an FCC phase with the unit cell parameter *a* = 4.032(4)Å, *V/Z* = 16.4 Å^3^, which corresponds to the specified composition of Ag_0.67_Pt_0.33_, considering the thermal expansion of metals.

### 2.7. Catalytic Properties

**Figure 10:**
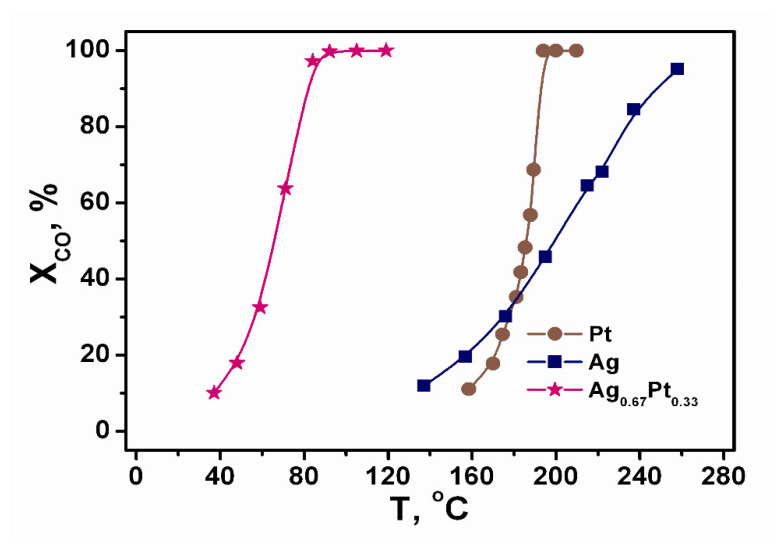
The temperature dependences of CO conversion (X_CO_) for CO TOX over the Pt, Ag, and Ag_0.67_Pt_0.33_ nano-powders. Feed gas composition (vol.%): 1.0 CO, 1.0 O_2_ with He as balance. WHSV: 80 000 cm^3^ g^−1^ h^−1^ (STP).

The catalytic properties of Ag_0.67_Pt_0.33_ nano-powder in CO TOX and PROX were compared with monometallic Pt and Ag nano-powders. The data on CO TOX is presented at Figure 10. It is observed that Ag activity is relatively low, reaching complete conversion of CO only at ca. 280 °C. It can be seen that over Pt, CO is oxidized at T = 160–200 °C. The addition of Ag significantly increases activity; a complete conversion of CO is already observed at 92 °C. As CO TOX over Pt is inhibited by dense surface coverage by adsorbed CO molecules [31], the positive effect of Ag doping could be associated with the realization of CO oxidation reaction at Ag–Pt interface sites. Adsorbed CO molecules over Pt atoms could react with oxygen atoms dissociatively adsorbed over Ag atoms. The presence of both Ag and Pt at essential quantities onto the surface of Ag_0.67_Pt_0.33_ was confirmed by XPS data (See Supplementary Materials). These results are in line with the literature data on advanced properties of Ag–Pt catalysts in CO TOX [15].

**Figure 11:**
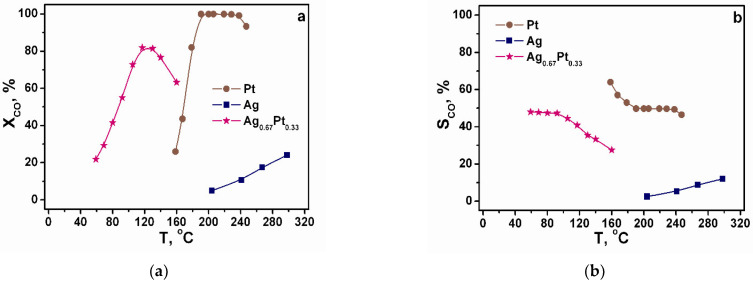
The temperature dependences of CO conversion (X_CO_) (**a**) and selectivity (S_CO_) (**b**) for CO PROX over the Pt, Ag, and Ag_0.67_Pt_0.33_ nano-powders. Feed gas composition (vol.%): 1.0 CO, 1.0 O_2_ with H_2_ as balance. WHSV: 80,000 cm^3^ g^−1^ h^−1^ (STP).

Nano-powders were studied in the CO PROX under reducing conditions. CO_2_ and H_2_O were the only products of the oxidation reactions of CO and H_2_, respectively. The formation of methane and other hydrogenation products was not observed. The obtained temperature dependences of CO conversion and selectivity are shown in Figure 11. It is seen that Ag is poorly active in CO PROX, predominantly catalysing H_2_ oxidation, and the oxidation of CO on pure Pt begins at T > ≈150 °C. In this case, the effect of blocking the Pt surface by adsorbed CO takes place. Complete conversion of CO is observed at temperatures of 190–240 °C. A further increase in temperature leads to an acceleration of the hydrogen oxidation reaction, which absorbs all the oxygen present in the system. Therefore, the decrease in CO conversion at high temperatures on Pt and other samples is due precisely to the competition between the CO and H_2_ oxidation reactions and the oxygen deficiency in the system.

Ag_0.67_Pt_0.33_ nano-powder is more active in CO PROX, starting oxidising CO at ≈50 °C, reaching the maximum conversion of 81% at 117–130 °C, and further oxidising predominantly H_2_ (Figure 11a). Properties of Ag_0.67_Pt_0.33_ with regard to CO oxidation at T < 100 °C under CO TOX and PROX conditions are close, indicating that the excess of H_2_ does not affect the catalyst performance at low temperatures while there is oxygen in reaction mixture. At the same time Ag_0.67_Pt_0.33_ oxidises H_2_ even at 50 °C, accelerating with increased temperature and becoming the predominant process which consumes all oxygen in the reaction mixture at T > 130 °C.

In summary, Ag_0.67_Pt_0.33_ is not a prospective catalyst for CO PROX despite high CO TOX activity due to high activity in the undesirable H_2_ oxidation reaction. This is in a good agreement with literature data as we have not found any information on good CO PROX performance of Ag–Pt alloy catalysts.

## 3. Materials and Methods

### 3.1. Synthesis of Initial Compounds and Nanoparticles

The source complexes H_2_[PtCl_6_] and K_2_[PtCl_6_] were synthesised following the method from [32]. All the initial reagents had ACS or AR purity grade.

The silver cation deposition reaction of the corresponding complex anion is used to obtain the Ag_2_[PtCl_6_] and Ag_2_[PtCl_4_] compounds. These compounds are synthesised with a high yield, which indicates their extremely low solubility. They do not dissolve in toluene, acetone or water.

Yield of Ag_2_[PtCl_6_] is 99%. Anal. Calc. for Cl6PtAg: (Pt + Ag), 65.89. Found: (Pt + Ag), 65.9.

Yield of Ag_2_[PtCl_4_] is 99%. Anal. Calc. for Cl4PtAg: (Pt + Ag), 74.34. Found: (Pt + Ag), 74.3.

Catalytic nanopowders of unordered solid solution Ag_0.67_Pt_0.33_ and Pt were prepared via Ag_2_[PtCl_6_] and [Pt(NH_3_)_4_](NO_3_)_2_∙2H2O salts decomposition in H_2_/He stream at 400 °C for 2 h.

### 3.2. Characterisation of Synthesised Substances

X-ray diffractometric analysis of the samples was performed on a DRON-RM4 diffractometer (Cu-*K_α_* radiation, a graphite monochromator using a reflected beam and a scintillation detector with amplitude discrimination, Burevestnik, Saint Petersburg, Russia). The samples were prepared by applying a suspension in alcohol on the polished side of a fused quartz cuvette. A polycrystalline silicon sample (a = 5.4309 Å) prepared in the same way was used as an external reference. The diffraction patterns were recorded in a step-by-step mode in 2*θ* angles range of 5°–120°.

X-ray phase analysis (XRD) of the thermolysis products was carried out in accordance with the data given in the PDF file for pure substances [33]. Parameters of the metal phases were refined over the entire data array using the Powder Cell 2.4 application programme [34]. The size of the crystallites in the obtained metal powders was estimated from the coherent scattering regions as a result of Fourier analysis of the profiles of single diffraction peaks using the WINFIT 1.2.1 software [35].

The composition of the obtained bimetallic solid solutions was determined using calibration curves of the volume per atom ratio (*V/Z*, where *V* is the volume of the unit cell, and *Z* is the number of structural units in it, that is atoms in this case) depending on the concentration of one of the metals. The calibration curves were plotted from the experimental values of atomic volumes for single-phase solid solutions of known composition given in references for Ag–Pt systems [36,37,38].

Thermogravimetric analysis (TGA) in inert and reducing atmospheres was performed using a NETZSCH TG 209 F1 Iris ^®^ thermal microbalance (Erich NETZSCH GmbH & Co. Holding KG, Selb, Germany). The sample weight was ~10 mg. Al crucibles were used, with the gas flow rate being 60 mL/min and the heating rate of 10 deg/min within the range 20–600 °C.

Differential scanning calorimeter NETZSCH 204 F1 Phoenix (Erich NETZSCH GmbH & Co. Holding KG, Selb, Germany) was used to study thermodynamic properties. DSC measurements were carried out by heat flow measurement method at a 6 K min^−1^ heating rate in 25 mL min^−1^ Ar flux in an unsealed aluminium crucible with lid. Powdered samples were distributed uniformly over the bottom, carefully tamped in aluminium crucibles. The sensitivity of the sample carrier sensors and temperature scale graduation were calibrated by melting and crystal-to-crystal transition measurements of standard samples (Hg, Ga, benzoic acid, KNO_3_, In, Sn, Bi, Pb, and Zn). The baseline signal obtained by heating two empty crucibles was subtracted from the experimental results of the samples. Netzsch Proteus Analysis software was used to determine DSC peak areas and transition temperature values. The transition temperature was defined from the resulting heat flow as intersections of the peak onset with the corresponding baseline. The peak area was determined by integrating the area between the measurement curve and the integral tangential baseline. The values of temperatures T_onset_ and enthalpy ΔH are the average of three measurements.

The disproportionation reaction of Ag_2_[PtCl_4_] on Ag_2_[PtCl_6_], Pt and AgCl was simulated in Jaguar 8.2, Schrödinger Inc. software [39] using the Density Functional Theory (DFT) and the Becke three-parameter hybrid functional (B3LYP) [40,41,42,43]. Relativistic effects were included by using effective core potentials (ECPs) for Pt and Ag. The LACVP+* [44] and LACV3P+* [44,45] basis sets associated with the ECPs were employed for Pt and Ag atoms, respectively, and 6–311G+* basis set [46,47] for Cl atoms.

Microscopic studies were carried out using an electron microscope JEM-2010 (with a resolution of 1.4 Å at an accelerating voltage of 200 kV, JEOL Ltd., Tokyo, Japan). Local energy-dispersive X-ray analysis (EDXA) was performed using an EDX spectrometer (EDAX Co., Tokyo, Japan) equipped with a Si(Li) detector with a resolution of 130 eV. The samples for the TEM study were prepared by ultrasonic dispersing in ethanol and consequent deposition of the suspension upon a “holey” carbon film supported on a copper grid.

The thermal decomposition of the complex salt was studied in the XRD mode in situ using an XRK-900 high-temperature reactor chamber (Anton Paar, Austria) mounted on an X-ray powder diffraction instrument in the precise diffractometry system at the Siberian Synchrotron and Terahertz Radiation Centre (SSTRC, Novosibirsk, Russia), and an OD-3M-350 one-coordinate detector [15]. The detector has 3328 channels covering the *2θ* angles range of 30°. The synchrotron radiation wavelength *λ* is 1.720 Å. XRD patterns were recorded for 60 s. Samples of the complex compound were loaded into an open holder that allowed gas (hydrogen) to pass through, and then the holder was placed in the reactor chamber.

### 3.3. Catalytic Testing

The CO PROX and TOX tests were performed in a fixed-bed continuous-flow quartz reactor (i.d.: 3 mm) at atmospheric pressure. A portion of 50 mg (particle size less than 50 μm) of the catalysts was placed in the reactor between two filters made of fused needle silica. The temperature was measured by a K-type thermocouple in the middle of the catalyst bed. The experiments were performed with the following feed gas composition (vol. %): 1.0 CO, 1.0 O_2_ with H_2_ as balance for CO PROX, and 1.0 CO, 1.0 O_2_ with He as balance for CO TOX, at WHSV of 80,000 cm^3^ g^−1^ h^−1^ (STP). Prior to the experiments, the samples were pre-treated in He flow for 1 h at 200 °C before CO TOX testing and in H_2_ flow for 1 h at 300 °C for CO PROX testing. Then, the samples were cooled down to 200 °C, He (H_2_) flow was substituted by the reaction feed and the samples were held for 1 h in the reaction mixture. Afterwards, several cycles of temperature decreasing/increasing were performed: all samples demonstrated reproducible catalytic characteristics.

The inlet and outlet concentrations were determined online using a Chromos GC-1000 chromatograph equipped with molecular sieve 5A and Porapak Q columns and with thermal-conductivity (TCD) and flame-ionisation (FID) detectors (Supplementary Materials, S1). The combination of a methanator (containing a reduced nickel catalyst) and the FID allowed highly sensitive analysis of CO and CO_2_. The relative errors of CO, CO_2_, and O_2_ concentrations determination were 0.5%, 0.5%, and 1%, respectively. CO_2_ and H_2_O were the only products of the reactions; no CH_4_ or other hydrocarbons were observed. In the course of experiments, the carbon balance was controlled with an accuracy of ±1%.

The catalyst performance was characterised by the CO conversion (XCO) and selectivity (SCO) in the case of CO PROX which were calculated by the following equations:

X_CO_ = ([CO]_inlet_ − [CO]_outlet_)/[CO]_inlet_ · 100%, (1)

S_CO_ = ½([CO]_inlet_ − [CO]_outlet_)/ ([O_2_]_inlet_ – [O_2_]_outlet_) · 100%, (2)

where [CO]_inlet_ and [O_2_]_inlet_ are the inlet concentrations of CO and O_2_, respectively; [CO]_outlet_ and [O_2_]_outlet_ are the outlet concentrations of CO and O_2_, respectively.

## 4. Conclusions

Thermolysis of Ag_2_[PtCl_6_] and Ag_2_[PtCl_4_] compounds in a helium atmosphere occurs in two stages. At the first stage, the compounds decompose to form platinum and silver chloride and release gaseous chlorine. At the second stage, silver chloride is sublimated, while platinum metal remains in the solid phase. In contrast to the thermolysis of Ag_2_[PtCl_6_], the thermal degradation of Ag_2_[PtCl_4_] at 350 °C is accompanied by disproportionation into Ag_2_[PtCl_6_], silver chloride, and platinum metal. This assumption is confirmed by the change in the reaction enthalpy in the temperature range of 345–375 °C and the corresponding calculations using the Density Functional Theory (DFT). The calculated enthalpy of the proposed reaction gives good agreement with the experiment (calc. 345 kJ/mol, found 366 kJ/mol).

Thermolysis of Ag_2_[PtCl_6_] and Ag_2_[PtCl_4_] compounds in a hydrogen atmosphere occurs in two poorly differentiated stages. At low temperatures (below 350 °C), the compounds decompose, and silver is reduced to a metallic state, while a single-phase solid solution of Ag_0.67_Pt_0.33_ is formed in ex situ. An increase in the final temperature of thermolysis to 500 °C initiates stratification of the resulting metastable solid solution.

In situ X-ray study of the Ag_2_[PtCl_6_] thermolysis in a hydrogen atmosphere indicates that AgCl is formed and metallic platinum is released at the first stage. In the case of further heating, AgCl is reduced to metal, with prolonged homogenisation and the formation of a single-phase solid solution of Ag_0.67_Pt_0.33_ at 614 °C.

The catalytic activity of the resulting nanoalloy Ag_0.67_Pt_0.33_ is studied in the reaction of CO total (TOX) and preferential (PROX) oxidation. Ag_0.67_Pt_0.33_ exceeds Pt and Ag nano-powders in activity in CO TOX, but was not selective in CO PROX. Further research could be directed to transition from nanopowders to supported Ag–Pt catalysts and better particle size control; this will help to boost catalytic activity.

## Data Availability

All data generated or analysed during this study are included in this published article and its Supplementary Information files.

## Supplementary Materials

The following are available online. S1: Gas chromatography of catalytic reaction products (GC); Figure S1: Example of the GC data for outlet composition at temperature of low CO conversion; S2: UV–Vis spectroscopy of Ag_2_[PtCl_6_] and Ag_2_[PtCl_4_]; Figure S2: Photoluminescence emission (PL) spectra of Ag_2_[PtCl_6_] and Ag_2_[PtCl_4_]; S3: Ex situ X-ray diffraction of Ag_2_[PtCl_4_] in a hydrogen atmosphere; S4: Ex situ X-ray diffraction of Ag_2_[PtCl_6_] in a helium atmosphere; S5: X-ray photoelectron spectroscopy (XPS); Table S1: Brief description of the studied samples; Table S2: Relative atomic concentrations of elements in the near-surface layer of the studied samples; Table S3: Binding energies (eV); Figure S3: The Pt*4f* and Ag*3d* spectra of the studied samples. [48,49,50,51,52,53,54,55,56,57,58,59,60,61,62] are cited in the Supplementary Materials.

## References

[1] Deng R., Xia Z., Sun R., Wang S., Sun G. (2020). Nanostructured ultrathin catalyst layer with ordered platinum nanotube arrays for polymer electrolyte membrane fuel cells. J. Energy Chem..

[2] Prodromidis G.N., Coutelieris F.A. (2020). Solid Oxide Fuel Cell systems for electricity generation: An optimization prospect. Renew. Energy.

[3] Wang H., An K., Sapi A., Liu F., Somorjai G.A. (2014). Effects of nanoparticle size and metal/support interactions in Pt-catalyzed methanol oxidation reactions in gas and liquid phases. Catal. Lett..

[4] Lefferts L., Van Ommen J.G., Ross J.R.H. (1987). The influence of hydrogen treatment and catalyst morphology on the interaction of oxygen with a silver catalyst. Appl. Catal..

[5] Yaroslavtsev A.B., Dobrovolsky Y.A., Shaglaeva N.S.E., Frolova L.A.E., Gerasimova E.V., Sanginov E.A. (2012). Nanostructured materials for low-temperature fuel cells. Russ. Chem. Rev..

[6] Ren L.P., Dai W.L., Yang X.L., Xu J.H., Cao Y., Li H., Fan K. (2005). Direct dehydrogenation of methanol to formaldehyde over pre-treated polycrystalline silver catalyst. Catal. Lett..

[7] Dai W.L., Cao Y., Ren L.P., Yang X.L., Xu J.H., Li H.X., Fan K.N. (2004). Ag–SiO_2_–Al_2_O_3_ composite as highly active catalyst for the formation of formaldehyde from the partial oxidation of methanol. J. Catal..

[8] Yang Z., Li J., Yang X., Wu Y. (2005). Catalytic oxidation of methanol to methyl formate over silver—A new purpose of a traditional catalysis system. Catal. Lett..

[9] Balbuena P.B., Callejas-Tovar R., Hirunsit P.D., De La Hoz J.M., Ma Y., Ramírez-Caballero G.E. (2012). Evolution of Pt and Pt-alloy catalytic surfaces under oxygen reduction reaction in acid medium. Top. Catal..

[10] Liu H., Yang J. (2014). Bimetallic Ag–hollow Pt heterodimers via inside-out migration of Ag in core–shell Ag–Pt nanoparticles at elevated temperature. J. Mater. Chem. A.

[11] He W., Wu X., Liu J., Zhang K., Chu W., Feng L., Xie S. (2010). Formation of AgPt alloy nanoislands via chemical etching with tunable optical and catalytic properties. Langmuir.

[12] Wisniewska J., Yang C.M., Ziolek M. (2019). Changes in bimetallic silver–platinum catalysts during activation and oxidation of methanol and propene. Catal. Today.

[13] Wisniewska J., Ziolek M. (2017). Formation of Pt–Ag alloy on different silicas–surface properties and catalytic activity in oxidation of methanol. Rsc Adv..

[14] Bauer U., Spath F., Dull F., Bachmann P., Steinhauer J., Steinrack H.P., Papp C. (2018). Reactivity of CO and C_2_H_4_ on Bimetallic Pt_x_Ag_1-x_/Pt (111) Surface Alloys Investigated by High-Resolution X-ray Photoelectron Spectroscopy. ChemPhysChem.

[15] Hwang S.Y., Zhang C., Yurchekfrodl E., Peng Z. (2014). Property of Pt–Ag alloy nanoparticle catalysts in carbon monoxide oxidation. J. Phys. Chem. C.

[16] Yao W., Jiang X., Li M., Li Y., Liu Y., Zhan X., Tang Y. (2021). Engineering hollow porous platinum-silver double-shelled nanocages for efficient electro-oxidation of methanol. Appl. Catal. B Environ..

[17] Cao J., Guo M., Wu J., Xu J., Wang W., Chen Z. (2015). Carbon-supported Ag@ Pt core–shell nanoparticles with enhanced electrochemical activity for methanol oxidation and oxygen reduction reaction. J. Power Sources.

[18] Semaltianos N.G., Chassagnon R., Moutarlier V., Blondeau-Patissier V., Assoul M., Monteil G. (2017). Nanoparticles alloying in liquids: Laser-ablation-generated Ag or Pd nanoparticles and laser irradiation-induced AgPd nanoparticle alloying. Nanotechnology.

[19] Xu H., Yan B., Li S., Wang J., Wang C., Guo J., Du Y. (2018). Facile construction of N-doped graphene supported hollow PtAg nanodendrites as highly efficient electrocatalysts toward formic acid oxidation reaction. Acs Sustain. Chem. Eng..

[20] Lv J.J., Feng J.X., Li S.S., Wang Y.Y., Wang A.J., Zhang Q.L., Feng J.J. (2014). Ionic liquid crystal-assisted synthesis of PtAg nanoflowers on reduced graphene oxide and their enhanced electrocatalytic activity toward oxygen reduction reaction. Electrochim. Acta.

[21] Kamat P.V. (2002). Photophysical, photochemical and photocatalytic aspects of metal nanoparticles. J. Phys. Chem. B.

[22] Shao T., Zhang Q., Li J., He S., Zhang D., Zhou X. (2021). AgPt hollow nanodendrites based on N doping graphene quantum dots for enhanced methanol electrooxidation. J. Alloys Compd..

[23] Bakar N.A., Abdullah N.A., Salleh M.M., Umar A.A., Shapter J.G. (2018). Optimum growth time in AgPt nanofern preparation for enhancement of surface-enhanced Raman scattering intensity. Adv. Nat. Sci. Nanosci. Nanotechnol..

[24] Bakar N.A., Abdullah N.A., Salleh M.M., Umar A.A., Shapter J.G. (2019). Effect of silver concentration towards formationof AgPt nanofernfilms as SERS substrates. Materials Science Forum.

[25] Mawarnis E.R., Ali Umar A., Tomitori M., Balouch A., Nurdin M., Muzakkar M.Z., Oyama M. (2018). Hierarchical bimetallic AgPt nanoferns as high-performance catalysts for selective acetone hydrogenation to isopropanol. Acs Omega.

[26] Breisch M., Grasmik V., Loza K., Pappert K., Rostek A., Ziegler N., Sengstock C. (2019). Bimetallic silver–platinum nanoparticles with combined osteo-promotive and antimicrobial activity. Nanotechnology.

[27] Wisniewska J., Guesmi H., Ziolek M., Tielens F. (2019). Stability of nanostructured silver-platinum alloys. J. Alloys Compd..

[28] Yılmaz V.T., Icbudak H. (1996). Thermal decomposition characteristics of ammonium hexachlorometallate (IV) complex salts of platinum metals. Acta.

[29] Shubochkin L.K., Sorokin L.D., Shubochkina E.F. (1975). On the thermal decomposition of palladates(II) and (IV) of alkali metals. Russ. J. Inorg..

[30] Thaddeus B.M. (1990). Binary Alloy. Phase Diagrams.

[31] Snytnikov P.V., Belyaev V.A., Sobyanin V.A. (2007). Kinetic model and mechanism of the selective oxidation of CO in the presence of hydrogen on platinum catalysts. Kinet. Catal..

[32] Chernyaev I. (1964). Synthesis of Complex. Compounds of Platinum Group Metals.

[33] (2014). Powder Diffraction File, PDF-2.

[34] Kraus W., Nolze G. (2000). POWDERCELL 2.4, Program. for the Representation and Manipulation of Crystal Structures and Calculation of the Resulting X-Ray Powder Patterns.

[35] Krumm S. (1996). An interactive Windows program for profile fitting and size/strain analysis. Mater. Sci. Forum.

[36] Schneider A., Esch U. (1943). Das System Silber-Platin. Ein Beitrag zur Frage der Spannungskorrosion. Z. Elektrochem. Angew. Phys. Chem..

[37] Yan X.S., Lin P., Qi X., Yang L. (2011). Finnis–Sinclair potentials for fcc Au–Pd and Ag–Pt alloys. Int. J. Mater. Res..

[38] Ebert H., Abart J., Voitlander J. (1983). Metastable solid solutions in Ag^®^ Pt alloys. J. Less Common Met..

[39] (2015). Jaguar, Version 8.2.

[40] Becke A.D. (1993). Density-functional thermochemistry. III. The role of exact exchange. J. Chem. Phys..

[41] Lee C., Yang W., Parr R.G. (1988). Development of the Colle-Salvetti correlation-energy formula into a functional of the electron density. Phys. Rev. B.

[42] Vosko S.H., Wilk L., Nusair M. (1980). Accurate spin-dependent electron liquid correlation energies for local spin density calculations: A critical analysis. Can. J. Phys..

[43] Stephens P.J., Devlin F.J., Chabalowski C.F., Frisch M.J. (1994). Ab initio calculation of vibrational absorption and circular dichroism spectra using density functional force fields. J. Phys. Chem..

[44] Hay P.J., Wadt W.R. (1985). Ab initio effective core potentials for molecular calculations. Potentials for K to Au including the outermost core orbitals. J. Chem. Phys..

[45] Rinaldo D., Tian L., Harvey J.N., Friesner R.A. (2008). Density functional localized orbital corrections for transition metals. J. Chem. Phys..

[46] Clark T., Chandrasekhar J., Spitznagel G.W., Schleyer P.V.R. (1983). Efficient diffuse function-augmented basis sets for anion calculations. III. The 3-21+ G basis set for first-row elements, Li–F. J. Comput. Chem..

[47] Frisch M.J., Pople J.A., Binkley J.S. (1984). Self-consistent molecular orbital methods 25. Supplementary functions for Gaussian basis sets. J. Chem. Phys..

[48] Scofield J.H. (1976). Electron Spectrosc. Relat. Phenomena.

[49] Shirley D.A. (1972). High-Resolution X-Ray Photoemission Spectrum of the Valence Bands of Gold. Phys. Rev. B.

[50] Fairley N. CasaXPS: Processing Software for XPS, AES, SIMS and More. www.casaxps.com.

[51] Tenney S.A., He W., Ratliff J.S., Mullins D.R., Chen D.A. (2011). Characterization of Pt–Au and Ni–Au Clusters on TiO2(110). Top. Catal..

[52] Steinrück H.-P., Pesty F., Zhang L., Madey T.E. (1995). Ultrathin films of Pt onTiO2(110): Growth and chemisorption-induced surfactant effects. Phys. Rev. B.

[53] Barr T.L. (1978). An ESCA study of the termination of the passivation of elemental metals. J. Phys. Chem..

[54] Bernsmeier D., Sachse R., Bernicke M., Schmack R., Kettemann F., Polte J., Kraehnert R. (2019). Outstanding hydrogen evolution performance of supported Pt nanoparticles: Incorporation of preformed colloids into mesoporous carbon films. J. Catal..

[55] Gołąbiewska A., Lisowski W., Jarek M., Nowaczyk G., Zielińska-Jurek A., Zaleska A. (2014). Visible light photoactivity of TiO2 loaded with monometallic (Au or Pt) and bimetallic (Au/Pt) nanoparticles. Appl. Surf. Sci..

[56] Smirnov M.Y., Vovk E.I., Nartova A.V., Kalinkin A.V., Bukhtiyarov V.I. (2018). An XPS and STM study of oxidized platinum particles formed by the interaction between Pt/HOPG with NO2. Kinet. Catal..

[57] Lamb R. (2004). Surface characterisation of Pd-Ag/Al2O3 catalysts for acetylene hydrogenation using an improved XPS procedure. Appl. Catal. A: Gen..

[58] Kaushik V.K. (1991). XPS core level spectra and Auger parameters for some silver compounds. J. Electron. Spectrosc. Relat. Phenom..

[59] Bukhtiyarov A.V., Stakheev A.Y., Mytareva A.I., Prosvirin I.P., Bukhtiyarov V.I. (2015). In situ XPS study of the size effect in the interaction of NO with the surface of the model Ag/Al2O3/FeCrAl catalysts. Russ. Chem. Bull..

[60] Wang H., Luo S., Li X., Liu W., Wu X., Weng D., Liu S. (2019). Thermally stable Ag/Al2O3 confined catalysts with high diffusion-induced oxidation activity. Catal. Today.

[61] Panafidin M.A., Bukhtiyarov A.V., Prosvirin I.P., Chetyrin I.A., Bukhtiyarov V.I. (2018). Model bimetallic Pd–Ag/HOPG catalysts: An XPS and STM study. Kinet. Catal..

[62] Glyzdova D.V., Afonasenko T.N., Khramov E.V., Leont’eva N.N., Prosvirin I.P., Bukhtiyarov A.V., Shlyapin D.A. (2020). Liquid-phase acetylene hydrogenation over Ag-modified Pd/Sibunit catalysts: Effect of Pd to Ag molar ratio. Appl. Catal. A Gen..

