# Supplementary material for: Formation of Catalytically Active Nanoparticles under Thermolysis of Silver Chloroplatinate(II) and Chloroplatinate(IV)
Source: Molecules. 2022 Feb 9;27(4):1173. doi: 10.3390/molecules27041173 (PMC8875358; doi:10.3390/molecules27041173)
Supplement: Supplementary file 1 [file molecules-27-01173-s001.zip › molecules-1548131-supplementary.pdf]

Formation of catalytically active nanoparticles under thermolysis of silver chloroplatinate(II) and chloroplatinate(IV)

E. Yu. Filatov[1,*], P. S. Smirnov[1,2], D.I. Potemkin[2,3], D.P. Pishchur[1], N.A. Kryuchkova[1], P.E. Plyusnin[1], S.V. Korenev[1]

[1]Nikolaev Institute of Inorganic Chemistry, Siberian Branch, Russian Academy of Sciences, Novosibirsk, 630090 Russia

[2]Novosibirsk State University, Novosibirsk, 630090 Russia

[3] Boreskov Institute of Catalysis, Siberian Branch, Russian Academy of Sciences, Novosibirsk, 630090 Russia

**e-mail: decan@niic.nsc.ru*

SUPPORTING DATA

S1 *Gas chromatography of catalytic reaction products (GC).*

The inlet and outlet concentrations were determined using a Chromos GC-1000 chromatograph equipped with molecular sieve 5Å and Porapak Q columns and with thermal-conductivity (TCD) and flame-ionization (FID) detectors. Molecular sieve column provided the separation of H_2, O_2, N_2, CO and CH_4 by residence time. Porapak Q column provided the separation of CO, CH_4, CO_2 and higher hydrocarbons by residence time. The combination of a methanator (containing a reduced nickel catalyst) and the FID allowed highly sensitive analysis of CO, CO_2 and hydrocarbons. In methanator all CO, CO_2, CH_4 and higher hydrocarbons molecules are converted in CH_4 by reaction with H_2 from added H_2 flow. Therefore they all gone to FID in the form of methane.

As an example, we presented the GC data for outlet composition at temperature of low CO conversion (Figure S1). It is seen, that no products were formed during the reaction except CO_2. (There are no peaks of CH_4, C_2H_4, C_2H_6 and etc.). The progress of the reaction is seen via the decrease of CO concentration and increase of CO_2 concentration (peak areas are proportional to the concentrations).

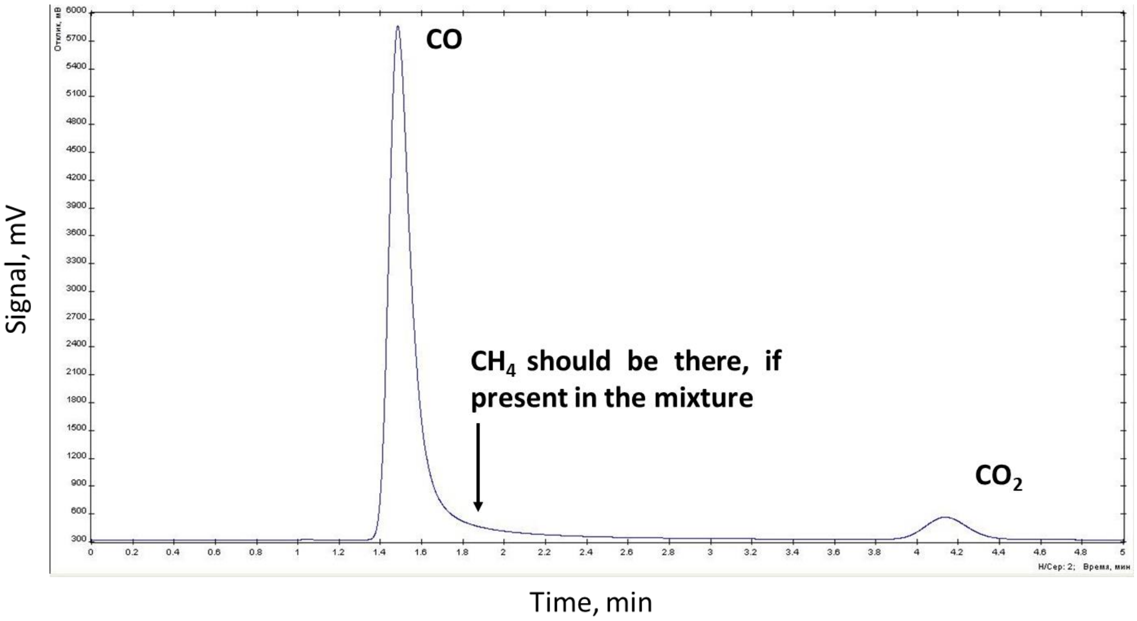

Figure. S1. Example of the GC data for outlet composition at temperature of low CO conversion

S2 *UV–Vis spectroscopy of $Ag_2[PtCl_6]$ and $Ag_2[PtCl_4]$*

UV–Vis spectra were recorded using a Shimadzu UV-3101PC spectrophotometer. The powder was placed in a Cu cell (ø 20 mm × 10 mm). The spectra were recorded in the range from 850 to 240 nm.

Photoluminescence emission (PL) spectra (Figure 2S) were recorded on a Varian Cary Eclipse fluorescence spectrometer with a 75 kW xenon light source (pulse length τ = 2 μs, pulse frequency ν = 80 Hz, wavelength resolution 0.5 nm; PMT Hamamatsu R928). Powder samples were placed in a cell equipped with a quartz window. PL spectra of both samples were measured under the same conditions.

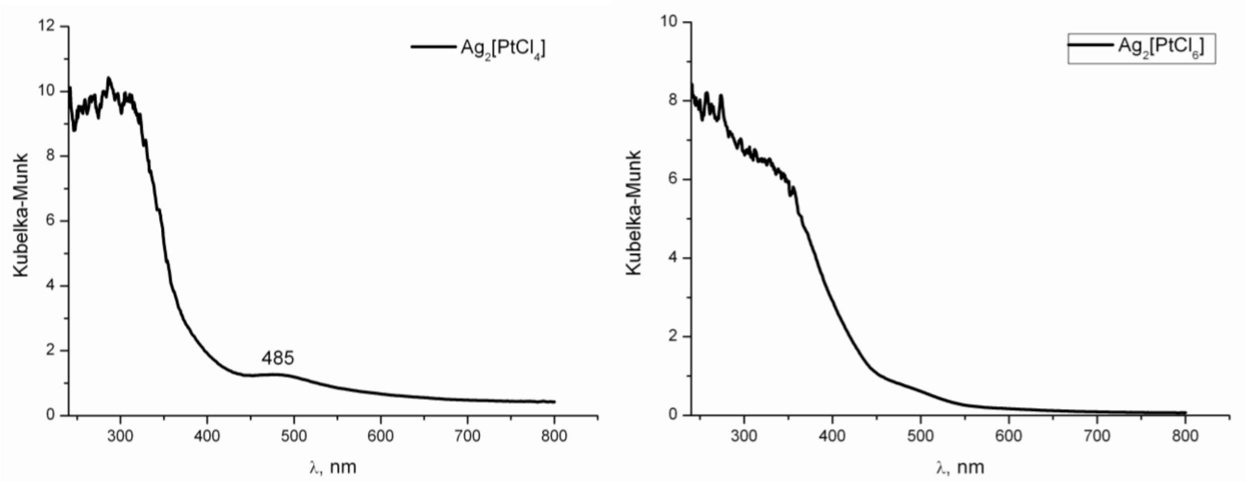

Figure. S2. Photoluminescence emission (PL) spectra of $Ag_2[PtCl_6]$ and $Ag_2[PtCl_4]$.

It can be seen that $Ag_2[PtCl_4]$ sample has an absorption at 485 nm. At the same time no emission was detected in the PL spectra. It means some chemical process is taking place such as

a silver reduction. Indeed the surface of the $Ag_2[PtCl_4]$ sample darkened after irradiation. However XRD pattern don’t show any phase changes. It means that photochemical reduction occurs only on the surface of the sample and amount of released silver is negligible for XRD analysis.

At the same time $Ag_2[PtCl_6]$ sample has no significant absorption and PL emission. The surface of the $Ag_2[PtCl_6]$ sample didn’t change color.

S3 *Ex situ X-ray diffraction of $Ag_2[PtCl_4]$ in a hydrogen atmosphere.*

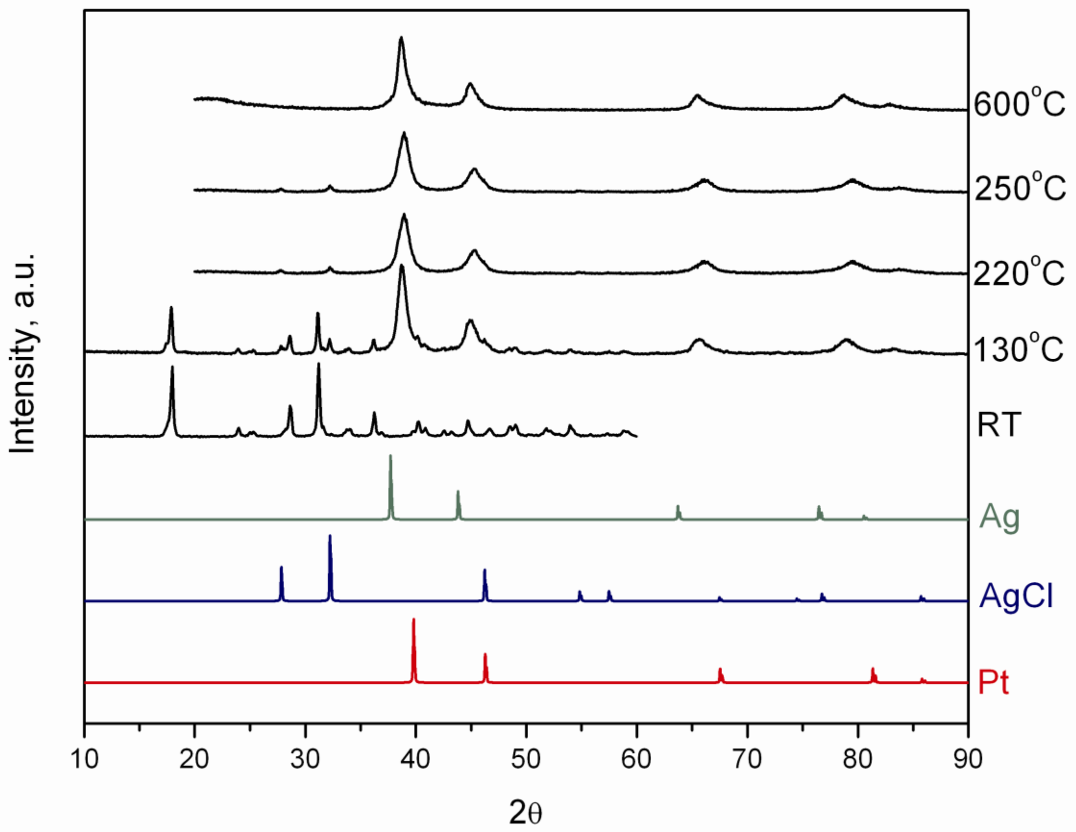

S4 *Ex situ X-ray diffraction of $Ag_2[PtCl_6]$ in a helium atmosphere.*

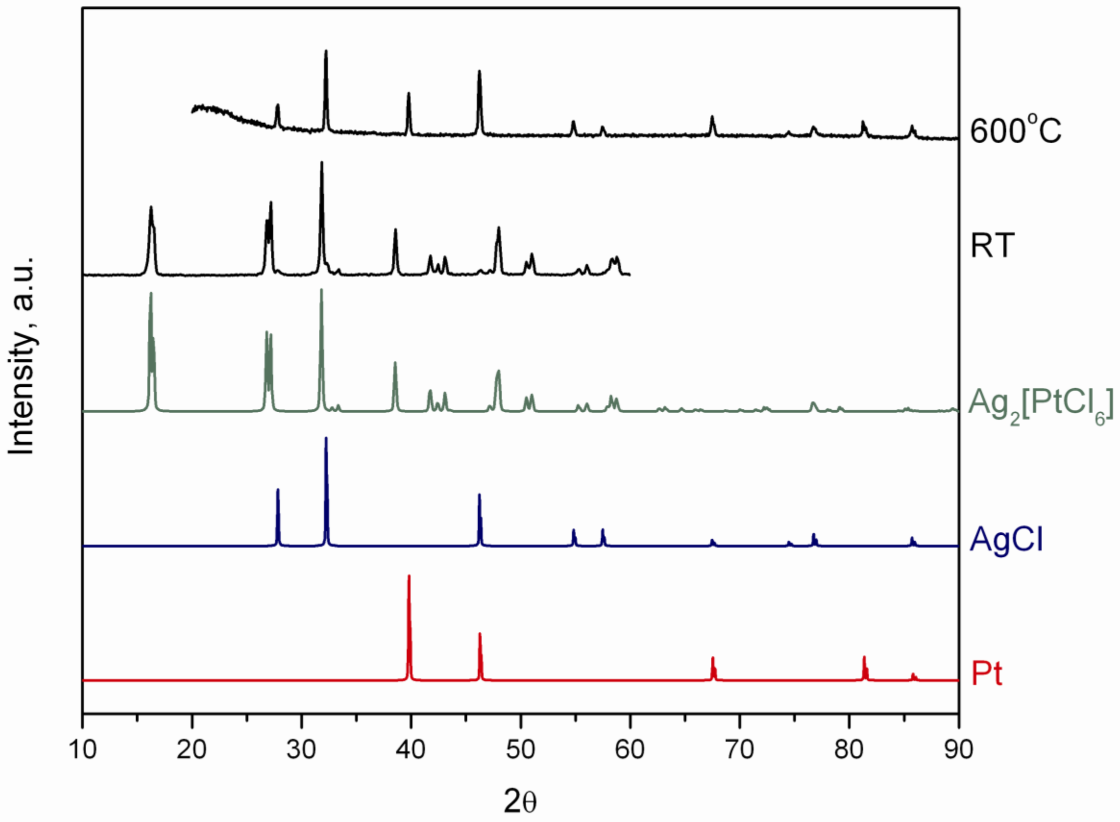

S5 *X-ray photoelectron spectroscopy (XPS)*

The study of the chemical composition of the samples surface was carried out on a SPECS Surface Nano Analysis GmbH (Germany) photoelectron spectrometer. The spectrometer is equipped with a PHOIBOS-150 hemispherical analyzer, an XR-50M X-ray source with a double Al/Ag anode and a FOCUS-500 monochromator. The spectra were recorded using monochromatic AlK_{α} ($h\nu$ = 1486.74 eV). To take into account the effect of sample charging, we used the position of the Fermi edge (E_F = 0.0 eV) in the valence band spectra. The relative concentrations of elements in the analysis zone were determined on the basis of the integral intensities of XPS lines taking into account the photoionization cross sections of the corresponding terms [48]. For a detailed analysis, the spectra were decomposed into individual components. Accordingly, after subtracting the background by the Shirley method [49], the experimental curve was decomposed into a number of lines corresponding to the photoemission of electrons from atoms in different chemical environments. Data processing was carried out using the CasaXPS software package [50]. The shape of the peaks was approximated by a symmetric function obtained by multiplying the Gauss and Lorentz functions.

Table S1. Brief description of the studied samples.

№	Sample	Composition	Initial salt
1	**sp098**	Ag_2Pt	Ag_2PtCl_4
2	**sp099**		Ag_2PtCl_6

In the XPS spectra of the samples, peaks corresponding to the chemical composition of the studied samples were found. The relative concentrations (atomic ratios) of elements in the near-surface layer of the samples, determined on the basis of XPS data, are presented in Table 2.

Table S2. Relative atomic concentrations of elements in the near-surface layer of the studied samples

№	Sample	[Pt]/[Ag]	[C]/[Ag]	[O]/[Ag]
1	**sp098**	0.28	2.88	0.58
2	**sp099**	0.28	5.73	1.09

Table S3. Binding energies (eV).

№	Sample	**Ag*3d*$_{5/2}$**	**Auger parameter α**	**Pt*4f*$_{7/2}$**	**C*1s***
1	**sp098**	367.7 (0.78)	725.6	70.3 (0.88)	284.8
2	**sp099**	367.7 (0.83)	725.6	70.3 (0.92)	284.8
3	**Ag foil**	368.3 (0.70)	726.2	–	
4	**Pt foil**	–		71.2 (0.82)	

The FWHM value for the corresponding peaks is indicated in brackets.

Figure S3 shows the Pt*4f* spectra of the studied samples, as well as the platinum foil. As is known, the *4f*-level of platinum, due to the spin-orbit interaction, splits into two sublevels Pt$4f_{7/2}$ and Pt$4f_{5/2}$, the spin-orbit splitting of which is 3.33 eV. The Pt*4f* spectrum of the studied samples and platinum foil is approximated by a single Pt$4f_{7/2}$–Pt$4f_{7/2}$ doublet with a Pt$4f_{7/2}$ binding energy in the region of 70.3 (for samples sp098 and sp099) and 71.2 eV (for platinum foil). According the literature, the Pt$4f_{7/2}$ binding energy of bulk samples of metallic platinum is 71.1–71.6 eV [51,52]. In turn, the Pt$4f_{7/2}$ binding energies of bulk PtO, PtO_2, and $Pt(OH)_4$ are in the range 72.3-73 eV, 74.0-74.1 eV and 74.2-74.4 eV [53–56]. Thus, this peak can be attributed to platinum in the metallic state; this statement is supported by the low value of the binding energy and the asymmetric shape of the peak. At the same time, the bond value is 0.9 eV less than that for bulk metallic platinum, which indicates the formation of an alloy with silver. It should be noted that, for platinum nanoparticles, with a decrease in the particle size, an increase in the Pt$4f_{7/2}$ binding energy occurs; the observed effect cannot be explained by the size of the metal particles.

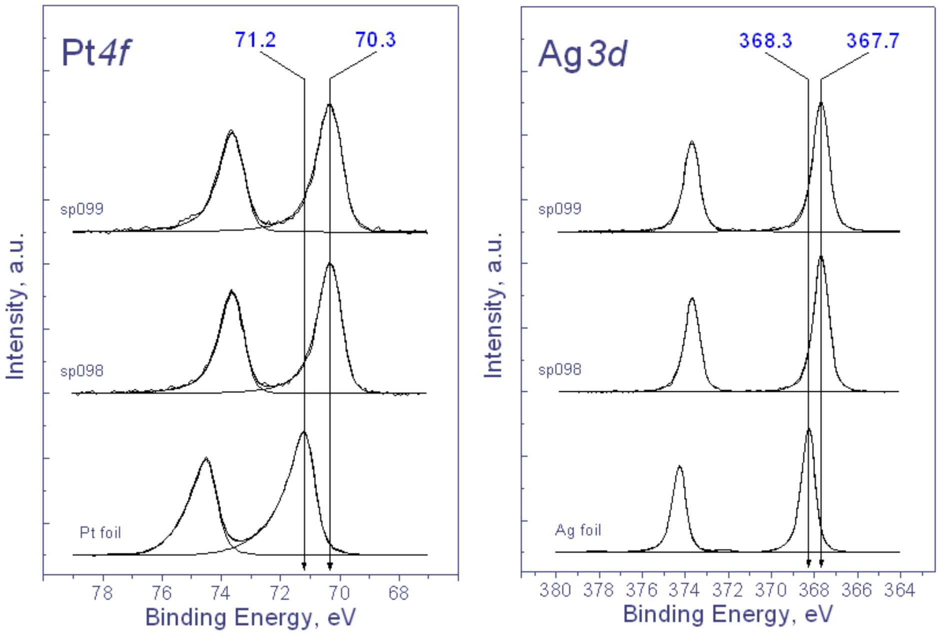

Fig. S3. The Pt*4f* and Ag*3d* spectra of the studied samples.

Figure 3S also shows the Ag*3d* spectrum of the studied samples, as well as the silver foil. As is known, the Ag*3d* spectrum is an Ag$3d_{5/2}$–Ag$3d_{3/2}$ doublet, the integral intensities of the components of which are in a ratio of 3:2. The spin-orbit splitting (the difference between the binding energies of Ag$3d_{5/2}$ and Ag$3d_{3/2}$) is 6.00 eV. In this case, the Ag*3d* spectra of the samples are well described by one doublet with the binding energy Ag$3d_{5/2}$ in the region of 367.7 eV, while the spectrum of bulk metallic silver is well described by one doublet with the binding energy Ag$3d_{5/2}$ in the region of 368.3 eV. The values of the Ag$3d_{5/2}$ binding energy of silver in different states change in a narrow range of 367.5-368.5 eV, which makes it difficult to identify the state of silver [57–62]. Nevertheless, the observed value of the binding energy unambiguously indicates that an alloy of silver and platinum is being formed. It should be noted that in some cases, to identify the state of silver, the so-called Auger parameter α is equal to the sum of the binding energy Ag$3d_{5/2}$ and the position of the maximum of the Auger spectrum of Ag*MNN* on the electron kinetic energy scale [57–62]. For all samples, the Auger parameter α is in the range of 725.6 eV, while for bulk silver this parameter is 726.2 eV, this fact also confirms the formation of a silver-platinum alloy.

[48] Scofield, J.H. Electron Spectrosc. *Relat. Phenom.* **1976**, 8, 129-137.

[49] Shirley, D.A., High-Resolution X-Ray Photoemission Spectrum of the Valence Bands of Gold. *Phys. Rev. B* **1972**, 5, 4709–4714.

[50] Fairley, N., CasaXPS: Processing Software for XPS, AES, SIMS and More. Available online : www.casaxps.com (accessed on 9 February 2022).

[51] Tenney, S.A., He, W., Ratliff, J.S., Mullins, D.R., Chen, D.A., Characterization of Pt–Au and Ni–Au Clusters on TiO_2(110), *Topics in Catalysis* **2011**, *54*, 42-55.

[52] Steinrück, H.-P., Pesty, F., Zhang, L., Madey, T.E., Ultrathin films of Pt onTiO_2(110): Growth and chemisorption-induced surfactant effects, *Physical Review B* **1995**, *51*, 2427-2439.

[53] Barr, T.L., An ESCA study of the termination of the passivation of elemental metals, *The Journal of Physical Chemistry* **1978**, *82*, 1801-1810.

[54] Bernsmeier, D., Sachse, R., Bernicke, M., Schmack, R., Kettemann, F., Polte, J., Kraehnert, R., Outstanding hydrogen evolution performance of supported Pt nanoparticles: Incorporation of preformed colloids into mesoporous carbon films, *Journal of Catalysis* **2019**, *369*, 181-189.

[55] Gołąbiewska, A., Lisowski, W., Jarek, M., Nowaczyk, G., Zielińska-Jurek, A., Zaleska, A., Visible light photoactivity of TiO_2 loaded with monometallic (Au or Pt) and bimetallic (Au/Pt) nanoparticles, *Applied Surface Science* **2014,** *317*, 1131-1142.

[56] Smirnov, M. Y., Vovk, E. I., Nartova, A. V., Kalinkin, A. V., Bukhtiyarov, V. I. An XPS and STM study of oxidized platinum particles formed by the interaction between Pt/HOPG with NO_2. *Kinetics and Catalysis* **2018**, *59*, 653-662.

[57] Lamb, R., Surface characterisation of Pd-Ag/Al_2O_3 catalysts for acetylene hydrogenation using an improved XPS procedure, *Applied Catalysis A: General* **2004**, *268*, 43-50.

[58] Kaushik, V. K. XPS core level spectra and Auger parameters for some silver compounds. *Journal of Electron Spectroscopy and Related Phenomena* **1991**, *56*, 273-277..

[59] Bukhtiyarov, A. V., Stakheev, A. Y., Mytareva, A. I., Prosvirin, I. P., Bukhtiyarov, V. I. In situ XPS study of the size effect in the interaction of NO with the surface of the model Ag/Al_2O_3/FeCrAl catalysts. *Russian Chemical Bulletin* **2015**, *64*, 2780-2785.

[60] Wang, H., Luo, S., Li, X., Liu, W., Wu, X., Weng, D., Liu, S. Thermally stable Ag/Al_2O_3 confined catalysts with high diffusion-induced oxidation activity. *Catalysis Today* **2019**, *332*, 189-194.

[61] Panafidin, M. A., Bukhtiyarov, A. V., Prosvirin, I. P., Chetyrin, I. A., Bukhtiyarov, V. I. Model bimetallic Pd–Ag/HOPG catalysts: an XPS and STM study. *Kinetics and Catalysis* **2018**, *59*, 776-785.

[62] Glyzdova, D. V., Afonasenko, T. N., Khramov, E. V., Leont'eva, N. N., Prosvirin, I. P., Bukhtiyarov, A. V., Shlyapin, D. A. Liquid-phase acetylene hydrogenation over Ag-modified Pd/Sibunit catalysts: Effect of Pd to Ag molar ratio. *Applied Catalysis A: General* **2020**, *600*, 117627.
